# A clue hidden in plain sight

**DOI:** 10.1093/ehjcr/ytag376

**Published:** 2026-05-21

**Authors:** Youmna Faheem, Shae Merves, Murad Almasri

**Affiliations:** Pediatrics Resident, BronxCare Health System Hospital, 1650 Grand Concourse, Bronx, NY 10457, USA; Pediatric Cardiology Department, Arkansas Children’s Hospital, 1 Children’s Way, Little Rock, AR 72202, USA; Pediatric Cardiology Department, Arkansas Children’s Hospital, 1 Children’s Way, Little Rock, AR 72202, USA

**Keywords:** Fascicular ventricular tachycardia, Verapamil-sensitive VT

## Clinical vignette

**Figure 1:**
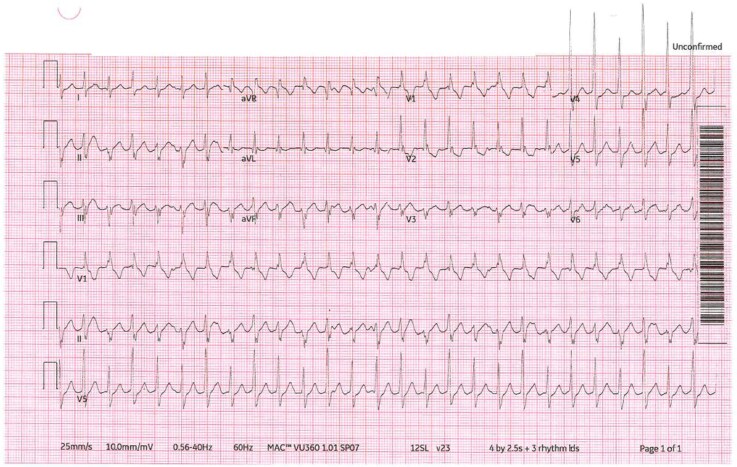
The patient's ECG that was obtained in the emergency department.

A 16-year-old female presented to the emergency room with palpitations. She has a history of previous normal ECGs and a normal echocardiogram. An ECG was done and showed the following (*Figure 1*). What is the diagnosis?

**1. What is the most likely diagnosis?**
  - Idiopathic ventricular fibrillation
  - Catecholaminergic polymorphic ventricular tachycardia
  - Belhassen ventricular tachycardia
  - Arrhythmogenic right ventricular cardiomyopathy
  - Supraventricular tachycardia with aberrancy

The correct answer is **C.**

Belhassen VT (idiopathic left fascicular VT) is a verapamil-sensitive ventricular tachycardia seen in young patients with structurally normal hearts, presenting as a relatively narrow complex tachycardia with right bundle branch block morphology and left axis deviation.^1^ Idiopathic ventricular fibrillation is incorrect as it is chaotic and not a stable monomorphic rhythm. Catecholaminergic polymorphic VT is incorrect because it is adrenergic-induced and polymorphic. Arrhythmogenic right ventricular cardiomyopathy is incorrect due to the absence of structural abnormalities. Supraventricular tachycardia with aberrancy is a mimic, but the QRS duration, axis, and morphology favour fascicular VT.

**2. What is the underlying electrophysiological mechanism responsible for this arrhythmia?**
  - Triggered activity due to delayed afterdepolarizations
  - Enhanced automaticity of Purkinje fibres
  - Reentry involving the left posterior fascicle and Purkinje network
  - Micro-reentry within scar tissue
  - Early afterdepolarization-mediated activity

The correct answer is **C.**

This arrhythmia results from a reentrant circuit involving the left posterior fascicle and Purkinje network,^2^ explaining its sensitivity to verapamil. Triggered activity (delayed afterdepolarizations) is incorrect as it is typically toxin- or catecholamine-related. Enhanced automaticity is incorrect because this is not a spontaneous focus. Scar-related micro-reentry is incorrect given the structurally normal heart. Early afterdepolarizations are incorrect as they are associated with prolonged QT and torsades de pointes.

**3. What is the most appropriate acute management for this condition?**
  - Intravenous amiodarone
  - Intravenous lidocaine
  - Electrical cardioversion
  - Intravenous verapamil
  - Beta-blocker administration

The correct answer is **D.**

Belhassen VT is uniquely sensitive to verapamil due to its dependence on calcium-mediated slow conduction in the reentry circuit.**^3^** Intravenous verapamil is the treatment of choice in stable patients. Other antiarrhythmics (e.g. lidocaine, amiodarone) are typically less effective, and cardioversion is reserved for haemodynamic instability. Beta-blockers are not first-line for this arrhythmia.

## Data Availability

No new data were generated or analysed in support of this research.

## Notes

**Consent:** The patient’s guardian consented to the publication of this case.
